# Single-Crystalline Gold Nanowires Synthesized from Light-Driven Oriented Attachment and Plasmon-Mediated Self-Assembly of Gold Nanorods or Nanoparticles

**DOI:** 10.1038/srep44680

**Published:** 2017-03-16

**Authors:** Shang-Yang Yu, Hariyanto Gunawan, Shiao-Wen Tsai, Yun-Ju Chen, Tzu-Chen Yen, Jiunn-Woei Liaw

**Affiliations:** 1 Department of Mechanical Engineering, Chang Gung University, Taiwan; 2 Graduate Institute of Biochemical and Biomedical Engineering, Chang Gung University, Taiwan; 3 Department of Orthopaedic Surgery, Chang Gung Memorial Hospital, Taiwan; 4 Center for Advanced Molecular Imaging and Translation, Chang Gung Memorial Hospital, Taiwan; 5 Department of Nuclear Medicine, Chang Gung Memorial Hospital, Taiwan; 6 Medical Physics Research Center, Institute for Radiological Research, Chang Gung University and Chang Gung Memorial Hospital, Taoyuan, 333, Taiwan; 7 Department of Mechanical Engineering, Ming Chi University of Technology, Taiwan

## Abstract

Through the light-driven geometrically oriented attachment (OA) and self-assembly of Au nanorods (NRs) or nanoparticles (NPs), single-crystalline Au nanowires (NWs) were synthesized by the irradiation of a linearly-polarized (LP) laser. The process was conducted in a droplet of Au colloid on a glass irradiated by LP near-infrared (e.g. 1064 nm and 785 nm) laser beam of low power at room temperature and atmospheric pressure, without any additive. The FE-SEM images show that the cross sections of NWs are various: tetragonal, pentagonal or hexagonal. The EDS spectrum verifies the composition is Au, and the pattern of X-ray diffraction identifies the crystallinity of NWs with the facets of {111}, {200}, {220} and {311}. We proposed a hypothesis for the mechanism that the primary building units are aligned and coalesced by the plasmon-mediated optical torque and force to form the secondary building units. Subsequently, the secondary building units undergo the next self-assembly, and so forth the tertiary ones. The LP light guides the translational and rotational motions of these building units to perform geometrically OA in the side-by-side, end-to-end and T-shaped manners. Consequently, micron-sized ordered mesocrystals are produced. Additionally, the concomitant plasmonic heating causes the annealing for recrystallizing the mesocrystals in water.

The mechanism of natural crystal growth has attracted a lot of attentions for centuries. The famous theory of Ostwald ripening, based on thermodynamics, has been used to explain the crystal growth^1^,^2^,^3^; nature prefers lower energy resulting in that smaller nanoparticles (solute) tend to dissolve and diffuse toward another larger one in a solution spontaneously. The well-known Ostwald ripening theory can be applied to explain the process of seed growth in ions solution for the formation of nanocrystals including nanoparticles (NPs) and even nanorods (NRs)^4^,^5^. Beside the theory of Ostwald ripening, recently another important theory of oriented attachment (OA) growth of nanocrystals has been proposed and recognized in the process of colloidal self-assembly, whereby adjacent NPs can combine with one another in a same crystal orientation to perform self-organization. The driving force of OA can be the van der Waals force, Coulombic (electrostatic) force, dipole-dipole interaction and so on^6^,^7^,^8^,^9^,^10^. This nonclassical crystal growth mechanism can successfully explain the mechanisms of the self-assembly synthesis of NPs, NRs and nanowires (NWs)^11^,^12^,^13^. However, because these driving forces are too weak and the distance of influence is too short, the probability of OA is very low. As a result, the natural OA processes always take a long time for nanoclusters to aggregate, align and form a large-sized crystal. Because of that, a few of methods using extreme conditions (high pressure^14^, higher temperature^15^), different types of irradiation energy (light^16^,^17^,^18^,^19^, two-photon^20^, electron beam^21^, X ray^22^) or surface tension (capillary force)^23^,^24^ have been proposed to enhance the OA process of NPs for forming a micron-sized crystal. On the other hand, the interplay of chemical and physical properties of the surface modification and medication (e.g. surfactant, ligand or DNA) was utilized to induce an attractive force for self-assembly of NPs^25^,^26^,^27^,^28^,^29^. For example, a variety of methods have been developed for orderly linking, organizing and assembling NRs in an end-to-end or side-by-side manner in water by functionalizing their surfaces with high affinity in the past decades^30^,^31^,^32^.

Among the various nanocrystals, Au and Ag NPs have attracted great attention, because of the surface plasmon resonance (SPR), which is a collective motion of free electrons corresponding to incident light. Comparted to Au NPs, Au NRs have an advantage of the tunable longitudinal SPR (LSPR) depending on its aspect ratio (AR)^33^,^34^. Recently, light-matter interaction was proven useful to the self-organization of colloidal Au and Ag NPs or NRs for forming certain patterns, e.g. 1D threaded chain; in particular the irradiation of femtosecond or nanosecond pulsed lasers can cause the sintering and welding of these NPs or NRs^35^,^36^,^37^. In addition, the light-induced oriented assembly of Ag NRs using a continuous-wave (CW) laser doughnut-beam with either azimuthal or radial polarization was studied^38^. Moreover, the plasmon-driven growths of Au nanoprisms and NRs using light-induced hot electrons interacting with surfactant or ions were proposed^39^,^40^. On the other hand, a variety of synthesis methods of Au or Ag NWs were proposed^41^,^42^,^43^,^44^,^45^,^46^. Several studies have pointed out that OA plays an important role for the formation of these NWs^13^,^47^. It was proven that the mechanical, electrical and optical properties of these metallic NWs depend on the crystallinity; normally single-crystalline Au NWs are superior to those of polycrystalline ones. For example, due to the high conductivity of single-crystalline Au NW, it can be utilized as an electrode to probe the neural signals of brain neurons^48^. Additionally, the attenuation of surface plasmon polariton along a polycrystalline Au NW is more severe than that along single-crystalline one^49^. This merit allows single-crystalline Au NWs can be applied for plasmonic waveguide of a photonic integrated circuit (PIC)^50^.

In this paper, we propose a new pathway of using optomechanics to induce the geometrically OA of Au NRs and NPs and the follow-up 3D self-assembly of the secondary and tertiary assemblies for the formation of anisotropic Au NWs. The experimental setup of the light-driven geometrically OA of Au NRs or NPs for the synthesis of Au NWs is shown in Fig. 1. Via the irradiation of a CW linearly polarized (LP) near-infrared (NIR) laser beam, the plasmon-mediated optical force and torque are induced upon coupled Au NRs or NPs for providing the directionality for the light-driven geometrically OA of these building blocks and the growth of Au NW, as well as the concomitant plasmonic heating is generated for the recrystallization of Au NW. Using the light-driven OA of primary building blocks (e.g. nanocrystals: NRs or NPs) to form larger structures, we can fabricate 3D micron-sized anisotropic mesocrystals and even single-crystalline Au NWs. Without any surfactant, ligand, DNA, polymeric binder or other additive, Au NWs can be fabricated in an aqueous solution at room temperature and atmospheric pressure by the irradiation of LP light alone. Herein, a new driving force, plasmon-mediated optical force and torque, is employed to induce the attraction and alignment for the geometrically OA of Au NRs or NPs and the subsequent 3D self-assembly of their secondary and tertiary assemblies (building units), instead of van der Waals force or electrostatic force (Coulomb interaction)^51^,^52^. The schematic illustration is shown in Fig. 2. The plasmon-enhanced polarizability of Au NRs (NPs) or even the assemblies facilitates the dipole-dipole interaction between adjacent NRs (NPs) or assemblies as irradiated by LP laser; the plasmon oscillation in each building unit is induced by LP laser to make it behave as an electric dipole. Additionally, the long axis of Au NW is the preferential direction for growing and supposed to be perpendicular to the light’s polarization^53^,^54^. Moreover, the concomitant plasmonic heating causes the recrystallization of the final assembly, leading to lattice reorganization. Consequently, single-crystalline Au NWs are fabricated. This simple method can be applied for the process of plasmonic waveguide in PIC or conducting channel in opto-electronic devices.

## Results

We used a CW LP NIR laser to irradiate a droplet of gold colloidal (Au NRs or NPs) suspension on a glass. Two types of Au NRs with different ARs were prepared; the LSPR of the first type is at 795 nm (AR = 4.18 ± 0.59), and that of the second type at 859 nm (AR = 6.5 ± 1.3); the images of transmission electron microscopy (TEM) and absorbance spectra of UV–vis–NIR spectrometer are shown in Fig. 3. The Au NPs with an average size of 48 nm with SPR peak at 537 nm were also synthesized for experiment. First, 1064-nm CW LP NIR laser beam was used to irradiate 13-ppm Au NR colloid with LSPR at 795 nm, where the fluence was 420 mW/cm^2^. The secondary building units resulting from the end-to-end, side-by-side or T-shaped OA of nearby Au NRs (primary building units) are observed in Fig. 4a. These secondary building units can be usually obtained at the beginning of irradiation or at the edge zone of laser beam with low irradiance. With an exposure time of 40 min, Au NR was produced in a droplet of 0.8 μl; Fig. 4b shows a FE-SEM image indicating one Au NR with pentagonal cross section accompanied with one Au NR. For our experience, single-crystalline Au NWs normally were synthesized in Au NR colloid irradiated by low-fluence laser beam within one hour. Figure S1 (in Supporting Information) shows a FE-SEM image of a lot of micron-length Au NWs synthesized in a 3- μl droplet with an exposure time of 60 min.

We also used 785-nm CW laser as light source to irradiate 10-ppm Au NR colloid with LSPR at 859 nm. Figure 5a shows FE-SEM image of single-crystalline Au NWs with average length of 4000 nm and width of 100 nm produced in a droplet of 0.8 μl irradiated by 785-nm CW laser beam with an average fluence (intensity) of 140 mW/cm^2^ for 40 min. The low-magnification FE-SEM image, as shown in Fig. S2, indicates this method can fabricate a lot of Au NWs (in Supporting Information). The high-magnification FE-SEM image is shown in Fig. 5b, which demonstrates that these Au NWs are with tetragonal cross section. In addition, the EDS was employed to analyze the composition of a single-crystalline Au NW. Figure 5c shows that these peaks in EDS spectrum correspond to gold atom indicating that the composition of this NW is gold alone. If the colloid was irradiated by a laser beam with a lower fluence of 70 mW/cm^2^, the hexagonal Au NW was synthesized, as shown in Fig. 5d; a droplet of 2 μl was irradiated by 785-nm laser beam for 40 min. Furthermore, the crystallinity of these Au NWs was examined by XRD, as shown in Fig. 5e. It can be seen that there are four distinct peaks of 2-theta at 38.3°, 44.6°, 64.3°, and 77.8°, corresponding to the facets of {111}, {200}, {220} and {311}, respectively^44^. These sharp narrow-band peaks indicate the high-degree crystallinity of these Au NWs. As is well known, gold crystal is a face-centered cubic (FCC) structure. As reported elsewhere, the required surface energies of the facets of FCC gold lattice obey {110} > {100} > {111}. Therefore, the annealing conditions are crucial for the formation of the single-crystalline Au NWs with different cross sections: tetragonal, pentagonal or hexagonal. Hence different laser irradiances generate different thermodynamic energy to tailor the shape of Au NW. In addition, after the laser is turned off, the cooling rate also affects the crystal growth rate to determine the shape. Since the plasmonic heating is generated locally in these assemblies, the cross-section shape of a single crystalline Au NW could be in relevance with the annealing and cooling conditions, depending on the laser power, exposure time, heating profile, concentration, volume of droplet, and laser wavelength. Due to the SPR the collective electron motion will induce Ohm loss in Au NP/NR to generate heat for annealing, which is the mechanism of plasmonic heating. According to our experience, a higher laser fluence associated with a slow cooling rate in nearly dried water at room temperature produces Au NWs of pentagonal shape, whereas a lower fluence with a faster cooling rate in water easily produces Au NWs of hexagonal and tetragonal shapes. Our finding is in agreement with the previous research; if the cooling is in air or nitrogen environment, the final cross section of recrystallized Au NW easily becomes pentagonal^55^. If the exposure time of laser is not long enough to provide the post heat treatment, only the polycrystalline mesocrystals are obtained (see Fig. S3 in Supporting Information). In contrast to the recrystallization temperature of bulk gold about 320 °C, the recrystallization temperature of Au nanostructures could be much low. Hence, the lattice reorganization and grain growth due to the mild annealing from the plasmonic heating inside the nanostructure could occur during the late stage of low-fluence laser irradiation. In general, the Au NWs with tetragonal and hexagonal cross sections are rarely synthesized by the other chemical methods, compared to the pentagonal one^45^. We can utilize this method to tailor the shape of NW by adjusting the heating profile. Additionally, through the light-driven end-to-end and side-by-side OAs of several Au NWs, an ultra-long NW can be self-assembled (see Fig. S4 in Supporting Information). Sometimes, the twisted micron-length Au belt can be produced by the light-driven method (see Fig. S5 in Supporting Information).

Although we do not have enough evidences yet, we propose a hypothesis for the light-driven OA that at the beginning the primary building units (Au NRs or NPs) are driven to perform geometrically OA by the optical force and torque induced by LP light to assemble and form larger secondary building units in an end-to-end or side-by-side manner for the subsequent self-assembly, and so forth the tertiary building units are formed. The schematic illustration of hypothesis is shown in Fig. 2. The plasmon-mediated optical forces and torques exerted upon these building units cause their translational and rotational motions in aqueous suspensions to perform the OA and 3D self-assembly. As a consequence, a micron-sized anisotropic mesocrystal is formed by the OA of Au NRs or NPs and 3D self-assembly of their secondary and tertiary building units; for example several secondary building unites (thin wires) can be bundled together, or few tertiary building united are aligned and coalesced. The probability of collision for the plasmon-mediated OA of adjacent building unites depends on the magnitudes of optical force and torque very much; these quantities are proportional to the fluence of laser beam. Therefore we can raise the laser power to increase the optical binding forces between adjacent NRs (or NPs) to increase the probability of plasmon-mediated collision. However, it also could cause more randomly orientated coalescence, which breaks the geometrically OA for the orderly arranged anisotropic pattern and induces more defects in the assemblies. Hence, a proper and moderate irradiance of laser is crucial for this process. Moreover, the successive plasmonic heating can sinter these building units to eliminate the defects at the junctions in the mesocrystal and make it recrystallized to form a single-crystalline Au NW. However, a severe heating could soften the structure to break the anisotropic shape. Under certain proper conditions, three types of Au NWs with different cross sections can be produced: tetragonal, hexagonal, and pentagonal, as shown in Fig. 2. The long axis of suspended Au NW is supposed to be perpendicular to the light’s polarization due to the optical torque^53^,^54^. This behavior is beneficial to the laser-assisted plasmonic heating of Au NWs^56^. However, the final orientations of Au NWs are not in the same direction. This is because that the capillary force changes their orientations as water is drying and the Brownian motion randomly alters the Au NW’s orientation after the laser is turned off. In fact, the surface tension (capillary force) also can be utilized to enhance the self-assembly and crystal growth of NPs for forming a micron-sized crystal by well controlling droplet evaporation^23^,^24^.

Sometimes, the branch Au NWs caused by the multiple twinnings in crystals can be obtained, as shown in Fig. 6a. If the laser power is raised to cause overheating, the temperature inside Au structure can be increased fast so as to soften the anisotropic assembly. Consequently, the 1D Au NW shrinks to break the anisotropic shape. For example, Au microcrystal of triangular dot is shown in Fig. 6b. According to the previous research, the Rayleigh-instability temperature (300 °C to 500 °C) of Au nanostructure is much lower than the melting point of bulk gold (1064 °C)^57^. In addition, since the fluence is not uniform in a Gaussian beam, a variety of nanostructures (NWs or triangular dots) can be produced at different locations in a bigger droplet (for the details, please see Fig. S6 in Supporting Information). If the laser power is large enough to melt the assembled Au structure, the dendritic or coralline structures could be produced during the solidification after the laser power is turned off. For example, Fig. 6c shows a coralline structure resulting from overheating^58^. The dendritic structure is shown in Fig. S7 due to the overheating (see Supporting Information)^59^. These cases illustrate that a low irradiance of laser to generate mild and modest plasmonic heating is of importance for the light-driven process of Au NW.

We also used Au NPs (diameter: 48 nm) as the building unites; a droplet of 3 μl containing 10-ppm Au NP colloid was irradiated by 785-nm LP laser beam. After a 10-min exposure with a fluence of 182 mW/cm^2^, the coalesced Au NPs-chain can be obtained, as shown in Fig. 7a. This demonstrates that 1D array assembly of Au NPs is feasible via the plasmon-mediated OA. As the exposure condition is 182 mW/cm^2^ for 20 min and then 336 mW/cm^2^ for 1 min, a single-crystalline Au NW (or block) with larger tetragonal cross section was obtained; the SEM image is shown in Fig. 7b. In general because the AR of Au NP is smaller than NR, the AR of the self-assembled anisotropic Au NW from Au NPs is less than that from NRs.

## Discussion

A method of using LP-light-matter interaction to fabricate single-crystalline Au NWs was proposed and demonstrated. The CW LP NIR lasers with low power were used as the light source, and the process was conducted in an aqueous droplet almost at room temperature and atmospheric pressure. The morphologies of single-crystalline Au NWs with tetragonal, pentagonal or hexagonal cross sections were verified by FE-SEM, and the crystallinity was identified the XRD pattern. This method is based on the plasmon-mediated OA of Au NRs or NPs (primary building blocks) and 3D self-assembly of the secondary, tertiary and even higher-order building units, as well as the plasmonic heat treatment for recrystallization. Through the plasmon-mediated optical forces and torques, the light-driven geometrically OA and 3D self-assembly of a large number of Au NRs or NPs in ordered organization becomes feasible. This light-driven OA method can bridge the gap of formation between nano- and micro-structure. Moreover, the concomitant mild plasmonic heating enables the post-recrystallization of the 3D anisotropic mesocrystals. As a result, single-crystalline Au NWs can be formed. Although the detailed mechanism is still not very clear so far, we propose the following hypothesis for that. During the process, the adjacent Au NRs or NPs are aligned and coalesced in the side-by-side or end-to-end manner to perform geometrically OA, driven by a LP laser beam. The plasmon-mediated optical forces and torques exerted upon adjacent Au NRs or NPs cause their translational and rotational motions to perform the geometrically OA in water. As a result, anisotropic-shaped structures are formed and guided by the polarization of light. Subsequently, the mild plasmonic heating at the adjacent NPs’ junctions causes the sintering and welding to form polycrystalline mesocrystals, and then induces annealing heat treatment to recrystallize the structures. Consequently, single-crystalline Au NWs are fabricated by the lattice reorganization. We believe that the whole plasmon-mediated process of single-crystalline Au NW involves multidisciplinary science, e.g. optomechanics, photophoresis and plasmonic annealing, worth further investigation. The LP light provides a shape-guiding force for the direction-selective growth of Au NW through the geometrically OA and self-assembly of Au NRs or NPs; the long axis of suspended Au NW is the preferential direction for growing, which is supposed to be perpendicular to the light’s polarization^53^,^54^. During the recrystallization, the surrounding water diffuses a part of heat accumulated in Au NWs to avoid the shrinking deformation resulting from overheating or melting. Additionally, water facilitates the translational and rotational mobility of building units for OA and 3D self-assembly. From the metallurgical thermodynamics viewpoint, the cross-sectional shapes (tetragonal, pentagonal and hexagonal) of Au NWs might depend on the annealing conditions (e.g. temperature, heating time and cooling rate). In addition to the plasmon-enhanced polarizability and plasmonic heating, the multifunctional performances of optical manipulation on Au NPs or NRs could include the photophoresis, which is worth exploring and exploiting further. The authors believe that the plasmon-mediated driving force induced by LP laser can accelerate the OA formation and 3D self-assembly of adjacent building unites to produce a single-crystalline Au NW, which is larger than the other driving forces, e.g. van der Waals force. The uniqueness of this method is that we can simultaneously adjust the optical driving force and torque for geometrically OA and the heat treatment for re-crystallization by tuning the laser power. In contrast to the natural OA of NPs due to the van der Waals forces but without post annealing, the light-driven method provides a pathway of geometrically OA of NRs (building blocks), dominated by the optical forces and torques rather than van der Waals forces. At the early stage of geometrically OA, the crystallographic directions of coalesced NRs/NPs at the junction are not necessarily the same. Nevertheless, the follow-up plasmonic heating will provide an annealing to recrystallize the assemblies for lattice reorganization, so that the orientations of lattices at the junction will be the same eventually, except the twinning. The advantages of this light-driven method of synthesizing single-crystalline NWs are fourfold. First, the process is without any other surfactant. Second, the plasmonic heating can provide a post recrystallization annealing for polycrystalline NWs fabricated by any other method (e.g. lithography) and retain their shapes. Third, the process time of this new method is within one hour. Forth, the process is conducted in water at room temperature and atmospheric pressure. In future, a beam shaper will be used to transform Gaussian beam into a uniform-intensity beam, which can facilitate the formation of Au NWs using light-driven geometrically OA. This new approach might pave the way to the bottom-up assembly of Au or Ag NPs and the associated heat treatment for fabricating micron-length single-crystalline NWs in the next-generation electronic nanocircuits of IC and plasmonic waveguides of PIC.

## Methods

**Figure 1:**
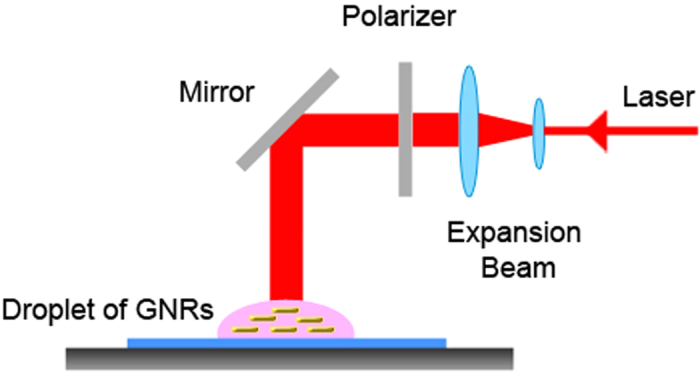
Experimental setup for the fabrication of single-crystalline Au NWs. A droplet of Au colloid on a glass is irradiated by a LP laser beam to induce plasmon-mediated OA of Au NRs or NPs.

**Figure 2:**
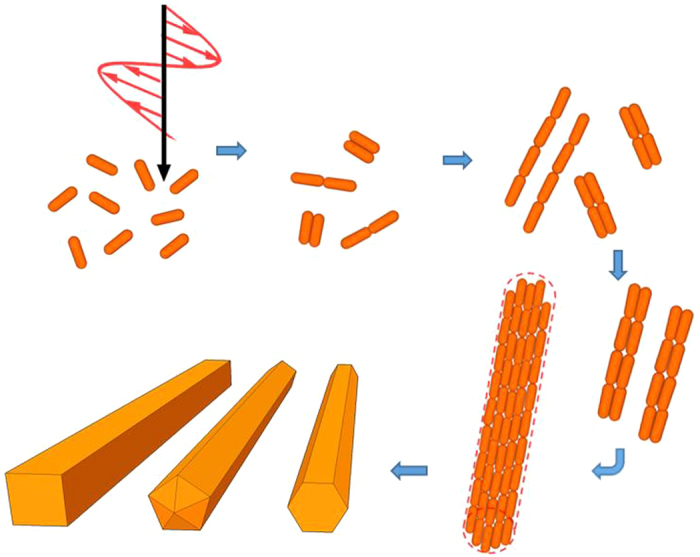
A hypothesis for the plasmonic-mediated synthesis of single-crystalline Au NWs. A schematic illustration for the light-driven formation of Au NWs including the plasmonic-mediated OA of Au NRs (primary building blocks) and 3D self-assembly of the secondary, tertiary and even higher-order building units, as well as the last stage of plasmonic heat treatment for recrystallization.

**Figure 3:**
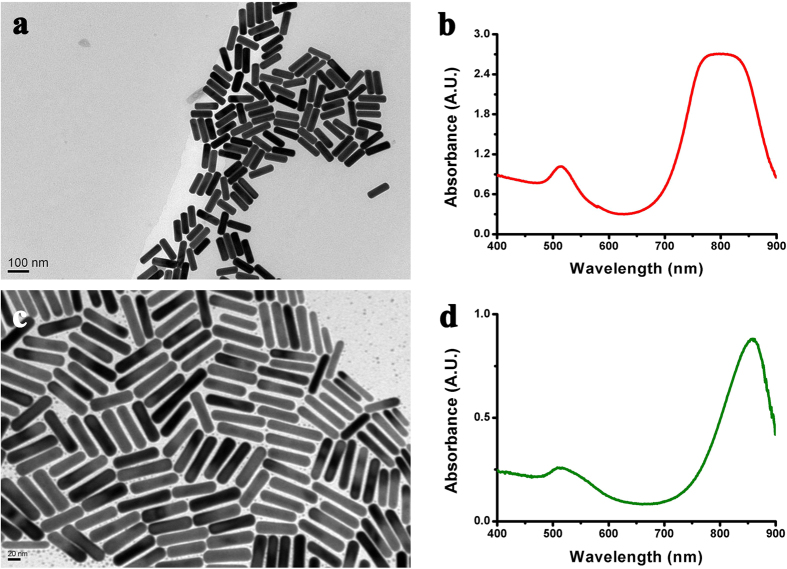
The morphologies and absorbance spectra of two types of Au NRs used as building units for the synthesis of NWs. (**a**) TEM image and (**b**) absorbance spectrum of Type-I Au NRs (LSPR: 795 nm, AR = 4.18 ± 0.59). (**c**) TEM image and (**d**) absorbance spectrum of Type-II Au NRs (LSPR: 859 nm, AR = 6.5 ± 1.3).

**Figure 4:**
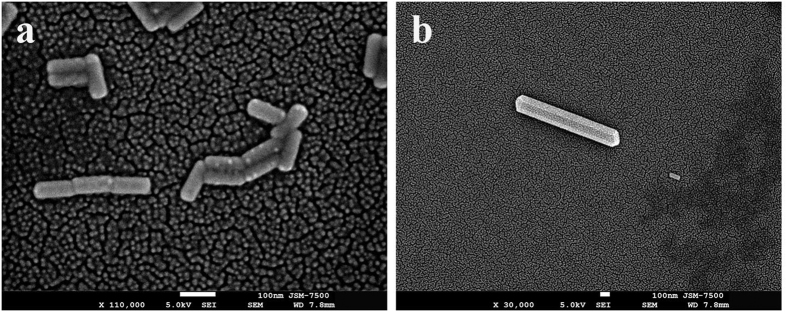
The plasmon-mediated self-assembly of Au NRs and the synthesized NW. (**a**) FE-SEM image of the secondary building units of the self-assembled Au NRs with end-to-end, side-by-side or T-shaped OA. (**b**) Single-crystalline Au NW with pentagonal cross section accompanied with Au NR.

**Figure 5:**
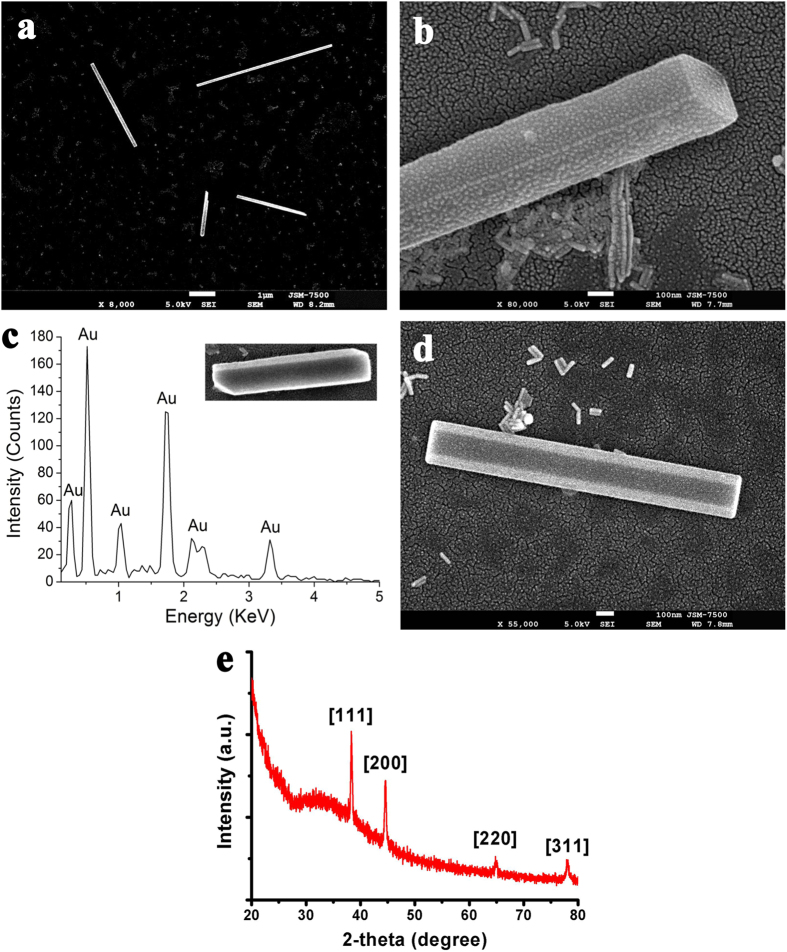
The single-crystalline Au NWs synthesized from the light-driven geometrically OA and plasmon-mediated self-assembly of Au NRs. (**a**) FE-SEM image of Au NWs with tetragonal cross section and (**b**) the high-magnification image. (**c**) EDS spectrum of Au NWs. (**d**) Au NW with hexagonal cross section. (**e**) XRD pattern of Au NWs.

**Figure 6:**
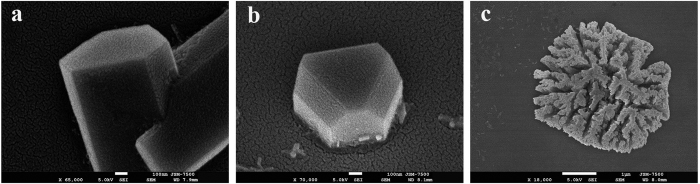
The other structures of plasmon-mediated self-assembly of Au NRs. (**a**) Branched Au NWs due to multiple twinnings. (**b**) 3D triangular dot due to the Rayleigh-instability caused by higher fluence. (**c**) Coralline structure due to overheating.

**Figure 7:**
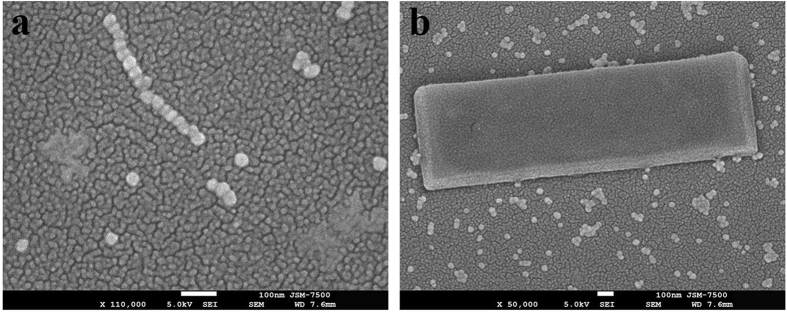
The plasmon-mediated self-assembly of Au NPs and the synthesized Au NWs. (**a**) Alignment and coalencence of 1D array Au NPs-chain (average diameter: 48 nm) induced by 785-nm LP laser irradiation for 10 min. (**b**) Au NW with tetragonal cross-section produced by an irradiation condition: fluence of 182 mW/cm^2^ for 20 min, and then 336 mW/cm^2^ for 1 min.

The experimental setup of the light-driven OA of Au NRs or NPs for the synthesis of Au NWs is shown in Fig. 1. A set of lenses was used to expand the laser beam. The beam size is about 3 mm for irradiation. If necessary, a polarizer was inserted into the optical path to polarize the laser beam. A power meter was used to measure the average fluence of the expanded laser beam. Because the fluence is not uniform in a Gaussian beam, we use the average fluence as the process condition in the following. These Au NRs of NPs were used as building blocks, and two CW LP NIR lasers (785 nm and 1064 nm) were used for experiment. The power of laser is around 3 mW to 30 mW; normally the average fluence for our experiments was less than 400 mW/cm^2^. In advance, Au NRs were synthesized by using chemical reduction method with binary surfactant mixture (CTAB-NaOL) as micelle-templates^34^. After the synthesis of Au NRs, we did our best to remove CTAB and NaOL by washing out the excess surfactants for several times. However, there could still be some tiny amount of CTAB and NaOL left on the surfaces of NRs. In fact, the residual CTAB and NaOL on the surfaces of NRs are useful to prevent the aggregation of NRs^60^. The concentrations of Au NR or NP colloids were measured by ICP-OES. For our experiments, the concentration of colloidal suspension is about 5 to 15 ppm, and the volume of a droplet is less than 10 μl. First, a droplet was placed on a glass, and then irradiated by CW LP laser beam. Before the droplet dried, the laser was turned off. We varied the laser power, wavelength, exposure time and concentration of Au NRs or NPs to investigate the feasibility of this photosynthesis of Au NWs. The wavelength range of LP light for this light-driven process is from red light to NIR. Actually, we have ever used CW lasers of 671, 785, 830 and 1064 nm for synthesis; all these lasers can help this process. Normally, the concentration of Au NRs/NPs should not be higher than 200 ppm, otherwise randomly aggregated assembles will be produced. We usually used 5 to 20 ppm of Au NRs/NPs for synthesis. The laser power should be less than 100 mW to avoid overheating, and the corresponding fluence is about 100 to 1000 mW/cm^2^. The volume of droplet is less than 3 μl. After the process, FE-SEM (JEOL JSM-7500F), energy dispersive spectroscopy (EDS) and X-ray diffraction (XRD) were employed to characterize the newly formed structures.

Notably, the water (surrounding medium) is necessary for the light-driven geometrically OA and plasmon-mediated self-assembly because that it facilitates the translational and rotational mobility of Au NRs or NPs (photophoretic moving and aligning) in the suspension to perform geometrically OA and 3D self-assembly. Moreover, due to the high specific heat (4.2 kJ/kg · K) water helps to diffuse the excessive heat accumulated in assemblies to avoid the overheating; the plasmonic heating resulting from the laser irradiation provides a mild annealing in water for the post heat-treatment of NW. During the process of Au NW, there is no sign of water boiling. However, increasing the laser power can raise the plasmonic heating rate in Au NR or NP colloid, leading to accelerating the droplet evaporation. The process of this physical method for colloidal self-assembly and coalescence is irreversible, which is entirely different from the other chemical methods using surfactant for templating or ligand/DNA for binding^29^. We believe that the whole plasmon-mediated process of single-crystalline Au NW involves multidisciplinary science, e.g. optomechanics, photophoresis and plasmonic annealing, worth further investigation.

## Additional Information

**How to cite this article:** Yu, S.-Y. *et al*. Single-Crystalline Gold Nanowires Synthesized from Light-Driven Oriented Attachment and Plasmon-Mediated Self-Assembly of Gold Nanorods or Nanoparticles. *Sci. Rep.* **7**, 44680; doi: 10.1038/srep44680 (2017).

**Publisher's note:** Springer Nature remains neutral with regard to jurisdictional claims in published maps and institutional affiliations.

## Supplementary Material

Supplementary Information
