# Supplementary material for: Single-Crystalline Gold Nanowires Synthesized from Light-Driven Oriented Attachment and Plasmon-Mediated Self-Assembly of Gold Nanorods or Nanoparticles
Source: Sci Rep. 2017 Mar 16;7:44680. doi: 10.1038/srep44680 (PMC5353694; doi:10.1038/srep44680)
Supplement: Supplementary Information [file srep44680-s1.pdf]

SUPPLEMENTARY INFORMATION

Single-Crystalline Gold Nanowires Synthesized from Light-Driven Oriented Attachment and Plasmon-Mediated Self-Assembly of Gold Nanorods or Nanoparticles

Shang-Yang Yu[1], Hariyanto Gunawan[1], Shiao-Wen Tsai[2,3], Yun-Ju Chen[1], Tzu-Chen Yen[4,5], Jiunn-Woei Liaw[1,4,6,7]*

[1]Department of Mechanical Engineering, Chang Gung University, Taiwan
[2]Institute of Biochemical and Biomedical Engineering, Chang Gung University, Taiwan
[3]Department of Orthopaedic Surgery, Chang Gung Memorial Hospital, Taiwan
[4]Center for Advanced Molecular Imaging and Translation, Chang Gung Memorial Hospital, Taiwan
[5]Department of Nuclear Medicine, Chang Gung Memorial Hospital, Taiwan
[6]Medical Physics Research Center, Institute for Radiological Research, Chang Gung University and Chang Gung Memorial Hospital, Taiwan
[7]Department of Mechanical Engineering, Ming Chi University of Technology, Taiwan

*Corresponding authors: markliaw@mail.cgu.edu.tw

Via the guiding of linearly polarized lights, multi-step and multifunctional optical manipulations on the growth of Au NWs from the geometrically orientated attachment (OA) and 3D self-assembly of the primary, secondary, tertiary and even higher-order building units seem to accomplish efficiently. Moreover, the successive recrystallization annealing of mesocrystals plays a crucial role in the process of single-crystalline Au NWs through the plasmon-mediated photothermal effect. Two types of Au NRs were prepared; the first type with LSPR at 795 nm (AR= 4.18), and the second type at 859 nm (AR= 6.5). **Figure S1** and **S2** show different Au NWs with pentagonal and tetragonal cross sections, respectively. **Figure S3** shows the FE-SEM images of polycrystalline mesocrystals for the cases without sufficient post heat

treatment of recrystallization. Sometimes, an ultra-long NW can be self-assembled through the light-driven end-to-end and side-by-side OA of several Au NWs, as shown in **Figure S4**. In addition, the twisted micron-length Au belt can be produced, as shown in **Figure S5**. Because the fluence is not uniform in a Gaussian beam, various nanostructures can be obtained in a bigger droplet. Normally, the triangular dots are easily produced at the center zone of laser beam, where the fluence is highest. **Figure S6** shows various structures produced at different locations in a bigger droplet. At the center zone of laser beam, dendrite Au structures are usually produced due to over-heating, as shown in **Figure S7**. This is because that the originally self-assembled nanostructure is molten and then rapidly solidified after the laser power is turned off.

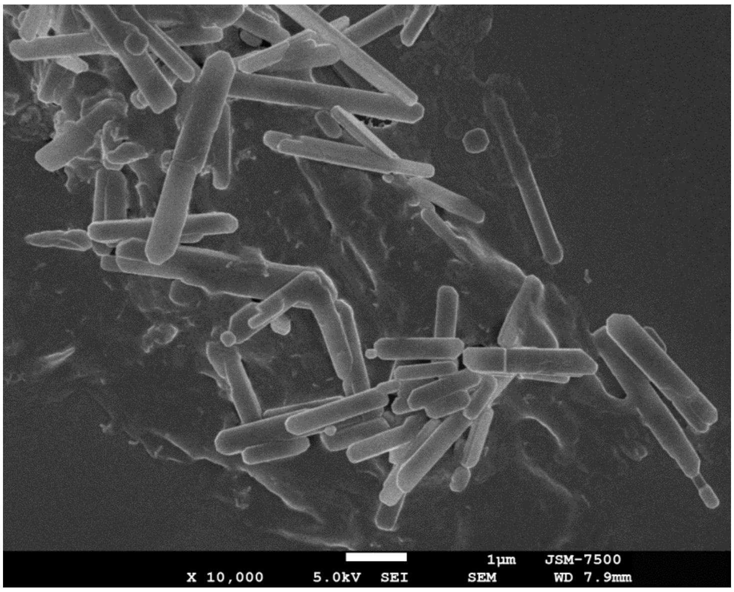

Figure S1. Micron-length anisotropic Au NWs. (3-μl droplet containing 13-ppm Au NR colloid with LSPR at 795 nm, irradiated by 1064-nm laser with average fluence of 420 mW/cm^2 for 60 min)

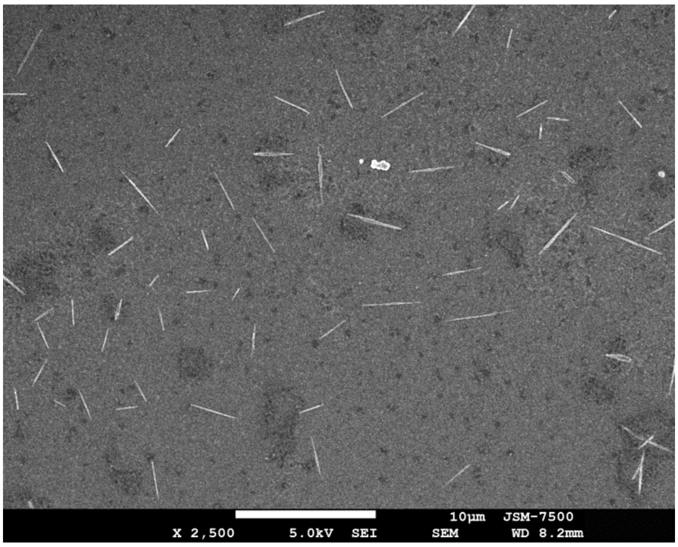

Figure S2. FE-SEM images of a lot of Au NWs with tetragonal cross section of low magnification (scale bar: 10 µm). (0.8-μl droplet of containing 10-ppm Au NR colloid with LSPR at 859 nm, irradiated by 785-nm laser with average fluence of 140 mW/cm^2 for 40 min)

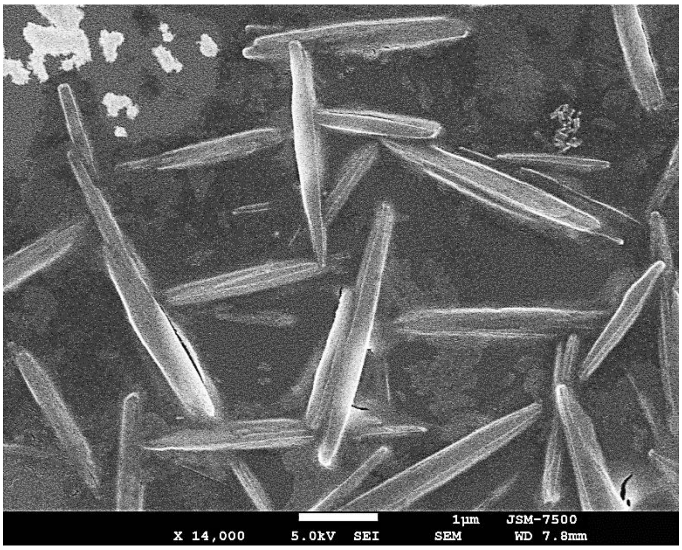

Figure S3. FE-SEM images of polycrystalline mesocrystals. (3-μl droplet of containing 10-ppm Au NR colloid with LSPR at 795 nm, irradiated by 1064-nm laser with average fluence of 70 mW/cm^2 for 20 min and then 140 mW/cm^2 for 2 min)

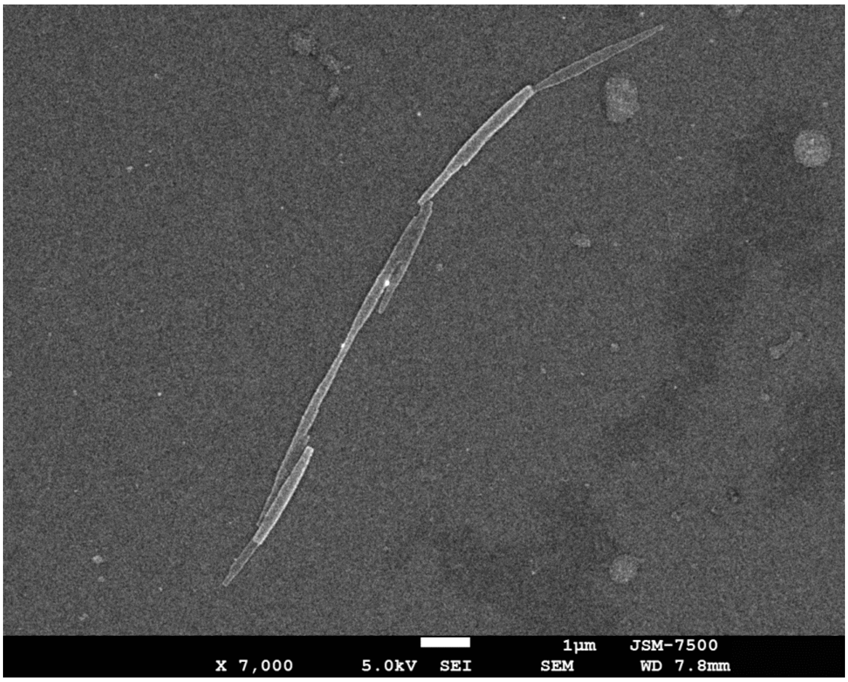

Figure S4. Ultra-long Au NW assembled by the end-to-end and side-by-side OA of several Au NWs (or tertiary building units) due to the optical forces and torques as well as the plasmonic coalescence. (3-μl droplet containing 10-ppm Au NR colloid with LSPR at 859 nm, irradiated by 785-nm laser with average fluence of 140 mW/cm^2 for 35 min, and then 280 mW/cm^2 for 3 min)

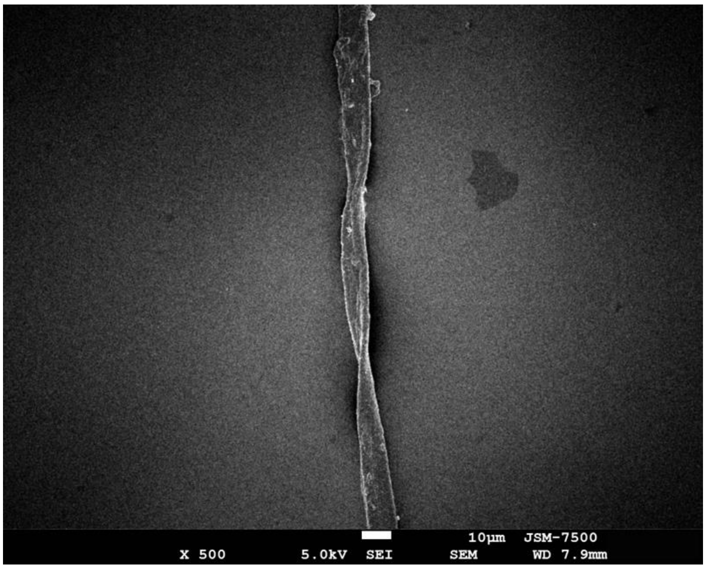

Figure S5. Twisted micron-length Au belt. (3-μl droplet of containing 10-ppm Au NR colloid with LSPR at 859 nm, irradiated by 785-nm laser with average fluence of 70 mW/cm^2 for 50 min)

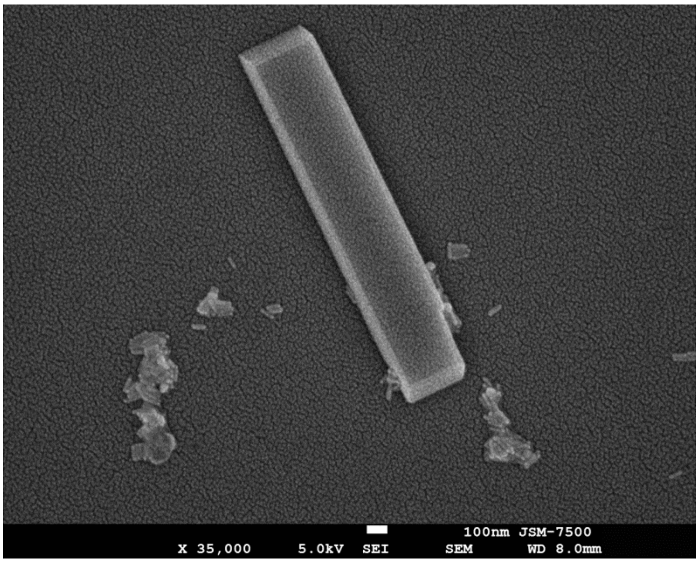
100nm JSM-7500
X 35,000 5.0kV SEI SEM WD 8.0mm

S6(a)

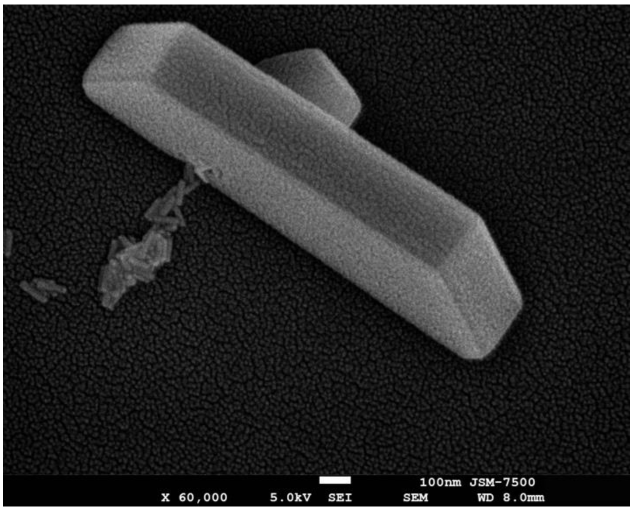
100nm JSM-7500
X 60,000 5.0kV SEI SEM WD 8.0mm

S6(b)

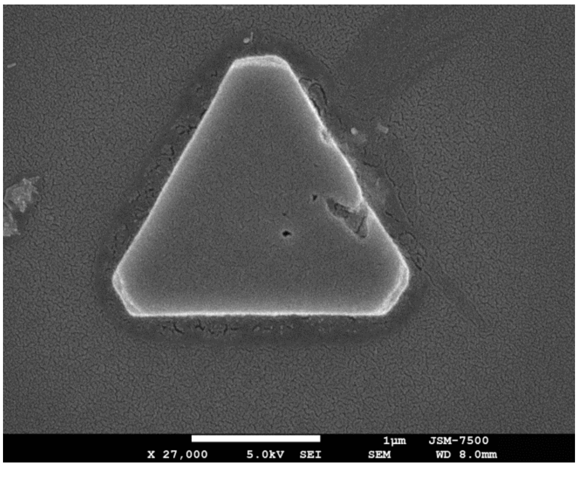
1μm JSM-7500
X 27,000 5.0kV SEI SEM WD 8.0mm

S6(c)

Figure S6. (a) Tetragonal Au NW with flat ends, (b) tetragonal Au NW with tapped ends and (c) triangular dot are produced at different locations in a bigger droplet of 3 μl with 10-ppm Au NRs (LSPR: 859 nm) irradiated by 1064-nm laser. Three steps with different irradiation conditions are used; average fluence: 280 mW/cm^2 for 15 min, fluence: 140 mW/cm^2 for 10 min, and then fluence: 280 mW/cm^2 for 5 min.

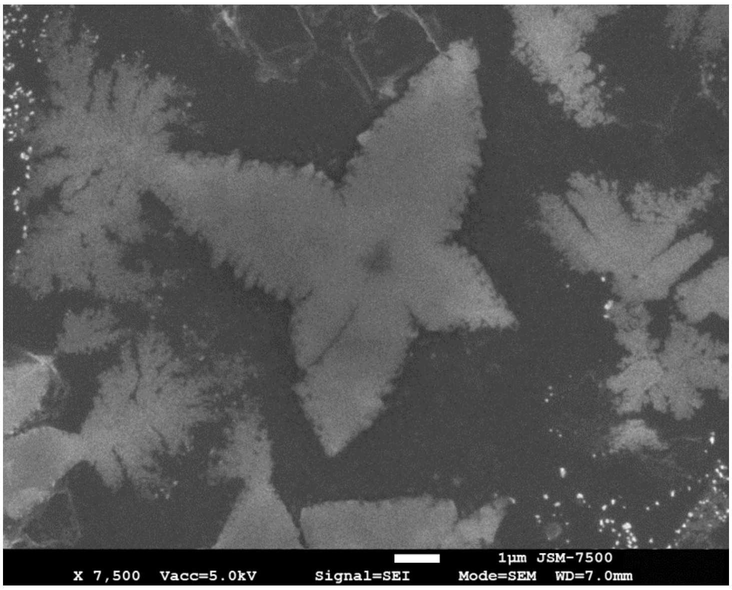

Figure S7. Dendrite Au structures are produced due to over-heating at the center zone of laser beam, where the fluence is highest. (3-μl droplet containing 10-ppm Au NR colloid with LSPR at 859 nm, irradiated by 785-nm laser with average fluence of 140 mW/cm^2 for 40 min)
